# Early biomarkers of acute kidney injury after lung transplantation

**DOI:** 10.1016/j.jhlto.2026.100679

**Published:** 2026-09-02

**Authors:** Vittorio Scaravilli, Alessandro Uslenghi, Gloria Turconi, Sebastiano Maria Colombo, Marco Bosone, Valentina Vago, Davide Pietro Carrà, Margherita Brivio, Letizia Corinna Morlacchi, Giuseppe Castellano, Alberto Zanella, Mario Nosotti, Lorenzo Rosso, Francesco Blasi, Giacomo Grasselli

**Affiliations:** a Department of Anesthesia, Critical Care and Emergency, Fondazione IRCCS Ca' Granda - Ospedale Maggiore Policlinico, Milan, Italy; b Department of Biomedical, Surgical and Dental Sciences, University of Milan, Milan, Italy; c School of Medicine and Surgery, University of Milan, Milan, Italy; d Department of Pathophysiology and Transplantation, University of Milan, Milan, Italy; e Thoracic Surgery and Lung Transplant Unit, Fondazione IRCCS Ca’ Granda - Ospedale Maggiore Policlinico, Milan, Italy; f Department of Internal Medicine, Respiratory Unit and Cystic Fibrosis Center, Fondazione IRCCS Ca' Granda - Ospedale Maggiore Policlinico, Milan, Italy; g Department of Nephrology, Fondazione IRCCS Ca' Granda - Ospedale Maggiore Policlinico, Milan, Italy

**Keywords:** Lung transplantation, Acute kidney injury, Biomarkers, Risk-stratification, Phenotypes

## Abstract

**Background:**

Acute kidney injury (AKI) is common after lung transplantation (LUTX), with standard diagnostic criteria having limited early accuracy. We evaluated the AKI and postoperative outcomes risk-stratification performance of subclinical AKI and creatinine-independent kidney-injury phenotypes.

**Methods:**

In this prospective single-center cohort including bilateral LUTX recipients, we measured urinary Tissue Inhibitor of Metalloproteinases-2 and Insulin-Like Growth Factor-Binding Protein 7 (u[TIMP-2]*[IGFBP-7]) and plasma Heart-Type Fatty Acid-Binding Protein (H-FABP), midkine, Soluble Tumor Necrosis Factor Receptor 1 (sTNFR1), and sTNFR2 at 6 and 36 hours after reperfusion, using point-of-care devices. AKI was defined by KDIGO criteria. Subclinical AKI was defined as u[TIMP-2]*[IGFBP-7]>0.3 without AKI criteria. Creatinine-independent phenotypes were identified with outcome-agnostic Latent Profile Analysis (LPA).

**Results:**

Among 84 included patients, 30 (36%) developed AKI. u[TIMP-2]*[IGFBP-7] had modest AKI discrimination, with the best AUROC 0.63 (95%CI 0.58-0.70) at 36 hours. Twenty-five patients had subclinical AKI, which had outcomes similar to those of patients without AKI. At 36 hours, plasma H-FABP, sTNFR1, and sTNFR2 were higher in patients with AKI. LPA identified 3 classes: low-risk (30%), intermediate-risk (51%), and high-risk (19%), with increasing rates of AKI (16%, 42%, 72%; *p* = 0.006) and primary graft dysfunction grade 3 at 72 hours (4%, 13%, 35%; *p* = 0.045). Compared with the low-risk class, the high-risk class had fewer organ support-free, ICU-free, and hospital-free days and the lowest rejection-free survival.

**Conclusions:**

After LUTX, u[TIMP-2]*[IGFBP-7] had limited risk-stratification performance, and subclinical AKI was not associated with a distinct clinical course. In contrast, plasma biomarker-derived risk phenotypes were associated with AKI severity and postoperative trajectory.

## Background

Lung transplant (LUTX) is the last therapeutic option for end-stage respiratory failure.^1^ Up to two-thirds of the LUTX recipients manifest post-operative acute kidney injury (AKI),^2^, ^3^, ^4^ as a result of ischemia-reperfusion,^5^ hypoxia, hemodynamic instability, inflammation,^6^ and direct glomerular or tubular damage (e.g., immunosuppressant and antibiotics).^7^ AKI is associated with a higher incidence of primary graft dysfunction (PGD), prolonged mechanical ventilation, and ICU stay.^8^ AKI frequently evolves into acute kidney disease (AKD)^4^ and chronic kidney disease (CKD),^9^ ultimately worsening the quality of life and long-term survival of LUTX recipients.^3^

The gold-standard diagnostic criteria for AKI—an increase in serum creatinine (sCr) or contraction of urinary output (UO)^10^—describe loss of kidney function and have limited early sensitivity. In LUTX recipients, the risk-stratification accuracy of sCr and UO is further limited by baseline patients' characteristics (i.e., muscle mass loss), intraoperative management (i.e., massive fluid resuscitation), and post-operative care (e.g., diuretics).

In diverse surgical settings, several emerging biomarkers for early AKI detection have been described.^11^, ^12^, ^13^, ^14^, ^15^ Recent guidelines suggest that patients with a biomarker positivity without standard criteria could be classified as having subclinical AKI and considered for targeted early management strategies.^16^ At present, the urinary cell-cycle arrest proteins Tissue Inhibitor Of Metalloproteinases-2 (TIMP-2) and Insulin-Like Growth Factor Binding Protein-7 (IGFBP-7), which signal tubular injury, are the only clinical-grade biomarkers for early diagnosis of subclinical AKI,^11^, ^15^ but have never been assessed after LUTX.

Recent work in surgical populations has shown that plasma biomarkers of cardiac injury (Heart-Type Fatty Acid–Binding Protein - H-FABP), ischemia/reperfusion injury (midkine), and inflammation (Soluble Tumor Necrosis Factor Receptors 1 and 2 - sTNFR1 and sTNFR2) have promising performance.^17^ We hypothesized that these plasma biomarkers may be useful in describing the multiple pathophysiological pathways leading to AKI after LUTX and that this biological heterogeneity may be captured by identifying biological risk phenotypes using an outcome-agnostic latent profile analysis (LPA).

Accordingly, in this prospective observational cohort study of adult LUTX recipients, we aimed at: 1) evaluate the performance of u[TIMP-2]*[IGFBP-7] in predicting AKI and define subclinical AKI after LUTX; 2) identify creatinine-independent kidney-injury risk phenotypes based upon plasma H-FABP, midkine, sTNFR1, and sTNFR2; and 3) explore their association with postoperative outcomes.

## Material and methods

### Study design

This prospective observational cohort study was approved by the local Institutional Ethics Committee (Milano Area 2, Milan, Italy, # 1183/2021), registered at clinicaltrial.gov (NCT05907434), and performed in accordance with the "Declaration of Istanbul on Organ Trafficking and Transplant Tourism" and the STROBE guidelines.^18^ Written informed consent was obtained from all study participants.

Adults undergoing bilateral LUTX at our Institution from June 1st, 2023, to June 30th, 2025, were considered for inclusion. Exclusion criteria were: 1) age < 18 years; 2) single LUTX; 3) combined solid organ transplantation; 4) re-transplantation. Perioperative management of LUTX recipients at our Institution is detailed in the Online Supplement (Additional Methods) and has been previously described.^4^, ^19^, ^20^, ^21^, ^22^, ^23^

### Data collection

We collected data relative to enlistment (age, gender, body mass index (BMI), estimated glomerular filtration rate—eGFR,^24^ preoperative Chronic Kidney Disease—CKD, defined as an eGFR<90 mL/min/1.73 m² comorbidities, Lung Allocation Score (LAS)^25^); surgery (immunosuppressive induction therapy, Extracorporeal Membrane Oxygenation (ECMO), blood components); and donor status (age and type of donor, type: Donor after Brain Death - DBD or Donor after Cardiac Death); Oto score^26^; warm and cold ischemia times; Ex-Vivo Lung Perfusion^27^ use).

sCr was measured at enlistment, before LUTX (i.e., 6 hours before surgery commencement), daily during ICU and hospital stay, at any ambulatory follow-up within 90 days of LUTX, and at 1 year after LUTX. UO and the need for renal replacement therapy (RRT) were assessed daily during the ICU and hospital stay. Moreover, daily urinary creatinine (uCr) concentration was measured during the ICU stay, and creatinine clearance (CrCl) was calculated as follows:

$$\mathrm{CrCl}\;(\mathrm{mL}/\min)=\frac{\mathrm{Cr}\times \mathrm{UO}}{\mathrm{Cr}\times 1440}$$

The worst CrCl was then assessed up to the 7th post-operative day. For patients treated with RRT, the last CrCl prior to RRT commencement was considered.

### Study measures

Urine and blood samples were collected at ICU admission (i.e., <6 hours from graft reperfusion) and at 36 hours after graft reperfusion. u[TIMP-2]*[IGFBP-7] was measured using a fluorescence-immunoassay point-of-care device (Nephrocheck test, BioMérieux Italia S.p.A., Firenze, Italy), with results reported as (ng/mL)^2^/1,000. Blood samples were collected in EDTA tubes, centrifuged (1,500 G for 15 min), and assayed for H-FABP, sTNFR1/2, and midkine with a real-time, fully automated, clinical-staff-operable immunoanalyzer (Multistat, Randox, Crumlin, UK). Treating clinicians were blinded to biomarker results.

### Endpoints

The primary endpoint was a composite of postoperative AKI OR death at 7 days after LUTX. AKI was defined according to kidney disease improving global outcomes (KDIGO)^28^ if sCr increased by ≥ 0.3 mg/dL at 48 hours OR ≥1.5 times compared to pre-operative within 7 days after LUTX. AKI was staged 1, 2, or 3 based on an increase in sCr to 1.5 to 1.9 times, 2 to 2.9 times, and >3 times baseline, respectively, ≤7 days from LUTX and/or having a UO <0.5 mL/kg/hr for 6 to 12 hours, <0.5 mL/kg/hr for >12 hours, <0.3 mL/kg/hr for ≥24 hours or anuria for ≥12 hours. Need for RRT was considered as AKI stage 3. Moreover, patients were defined as having subclinical AKI if u[TIMP-2]*[IGFBP-7] was > 0.3 at any time point, in the absence of concomitant KDIGO-defined AKI.^29^

Secondary outcome measures included: 1) post-operative AKD^30^ stage 1, stage 2, and stage 3 whether sCr increased 1.5 to 1.9 times, 2 to 2.9 times, and >3 times (or need for dialysis) during the 90-day follow-up; 2) CKD at 1 year after LUTX,^24^ classified according to the 1-year eGFR G1 (≥90), G2 (60-89), G3 (30-59), G4 (15-29), and G5 (<15 mL/min/1.73 m^2^, chronic RRT, or death). Further, non-directly renal outcomes were assessed: PGD^31^ grade 3 at 72 hours post-reperfusion; duration of invasive mechanical ventilation; 28-day ventilation-free days; 28-day intensive care unit (ICU)-free days; vasoactive support duration; 28-day vasoactive-free days; and 60-day hospital-free days, biopsy-proven acute rejection at 6 months,^32^ and rejection-free survival on May 1st, 2026.

### Statistical analysis

Sample size was calculated to detect an area under the receiver operating curve (AUROC) of 0.70 for u[TIMP-2]*[IGFBP-7] in detecting AKI (stage 0 vs stage >0), assuming a null AUROC of 0.5, and a ratio of sample size in negative/positive groups (patients with AKI stage 0 /AKI stage >0 = 1.3) according to priors from our center.^4^ With α=0.05 and power 85%, the analyses yielded an estimation of a total sample size of 67 patients (Medcalc 20.015, MedCalc Software Ltd, Ostend, Belgium). To account for potential non-evaluable cases due to missing or lost biomarker measurements, the planned sample size was set at 85 patients.

Continuous variables are reported as median [25th-75th percentile], and categorical variables as the number (%). Comparisons between patients' cohorts were performed using chi-square, Fisher's, or the Wilcoxon rank-sum tests, as appropriate. For binary outcome measures, odds ratios (OR) and associated 95% likelihood ratio-based confidence intervals (95%CI) were calculated. For continuous non-parametric outcome measures, Vargha and Delaney's A or Cohen's D effect size estimates were calculated. Kaplan-Meier survival curve analysis was used with log-rank testing for time-to-event comparisons, with right censoring applied.

Biomarker concentrations were log-transformed and z-scores standardized. Missing biomarker values were not imputed. Associations between single biomarkers and AKI were analyzed using a nominal logistic regression at both time points. Separate models were fitted to assess the association of u[TIMP-2]*[IGFBP-7], plasma H-FABP, midkine, sTNFR1, and sTNFR2 with AKI. Predicted probabilities were used to construct AUROCs, with corresponding sensitivity, specificity, predictive values, and likelihood ratios.

LPA was performed on plasma concentrations of H-FABP, midkine, sTNFR1, and sTNFR2, using Gaussian finite mixture models. Models with 1–4 classes were estimated assuming diagonal covariance matrices with equal variance across classes. The number of latent classes was evaluated by fitting models with 1 to 4 classes. Model selection was based on the combined assessment of the Bayesian Information Criterion (BIC), Akaike Information Criterion, entropy, class size, posterior classification probabilities (maximum a posteriori assignment), and biological interpretability. Individuals were assigned to the class corresponding to their highest posterior probability (maximum a posteriori assignment).

As an exploratory extension, the final LPA solution selected according to the prespecified model-selection criteria was used to derive an individual-level phenotype classifier (see Online Supplement, Additional Methods). For a new patient, biomarker concentrations were transformed and standardized using the preprocessing parameters of the derivation dataset, and the fixed parameters of the selected mixture model were applied to estimate posterior probabilities of membership in each latent class. Patients were assigned to the class with the highest posterior probability. No model refitting was performed.

To assess the robustness of the clustering structure, an alternative unsupervised clustering approach (i.e., Hierarchical Cluster Analysis - HCA) using Ward’s method was performed on the same log-transformed and standardized biomarkers (see Online Supplement, Additional Methods).

Statistical significance was accepted at *P* < 0.05, and no correction for multiple comparison was performed, given the exploratory nature of the study. Analyses were performed using JMP Pro 18 (SAS Institute, Cary, NC) and R (R version 4.5.2), with the “mclust” package version 6.1.2.

## Results

### Study population

From June 1st, 2023, to June 30th, 2025, 97 patients underwent LUTX at our Institution. After excluding 12 patients, 85 met the inclusion criteria (see Figure S1, Additional Results, Online Supplement). One patient was not recruited due to logistical constraints, leaving 84 patients in the final analysis.

Table 1 summarizes patients’ baseline characteristics. Median pre-operative eGFR was 102.3 [95.8-110.9] (mL/min/1.73 m^2^), with 21% of the patients having pre-operative CKD stage>0. Thirty patients (36%) developed AKI. Pre-operative and intraoperative characteristics were largely comparable between AKI and non-AKI patients.

**Table 1:** Patients' Characteristics

		Overall (*n* = 84)	AKI=0 (*n* = 54)	AKI>0 (*n* = 30)	*p*
Sex (Female)		35 (42%)	21 (39%)	14 (47%)	0.489
Age at LUTX (years)		56 [45-62]	53.5 [44.75-61.0]	59.0 [44.5-62.2]	0.392
BMI (kg/m^2^)		23.2 [19.1-26.4]	22.9 [19.0-26.0]	24.5 [19.8-27.7]	0.239
Diagnosis	Interstitial Lung Disease	46 (55%)	27 (50%)	19 (63%)	0.219
	CF/Bronchiectasis	21 (25%)	16 (30%)	5 (17%)	
	COPD	10 (12%)	8 (15%)	2 (7%)	
	Other	6 (8%)	3 (6%)	3 (10%)	
	Vascular	1 (%)	0 (0%)	1 (3%)	
Comorbidities	Hypertension	26 (31%)	19 (35%)	7 (23%)	0.254
	Diabetes	13 (15%)	9 (17%)	4 (13%)	0.682
	CKD*	18 (21%)	8 (15%)	10 (33%)	0.051
	Hepatopathy	4 (5%)	4 (7%)	0 (0%)	0.056
Lung Allocation Score		41.6 [37.1-50.1]	41.5 [36.8-49.4]	43.0 [37.5-50.9]	0.698
Waiting List (days)		118 [47-315]	136 [45-366]	96 [46-242]	0.447
eGFR (mL/min/1.73 m^2^)		102 [94-111]	102 [97-111]	98 [85-94]	0.144
ECMO bridge to LUTX		2 (2%)	2 (4%)	0 (0%)	0.180
Age (years)		52 [32-58]	53 [32-57]	51 [33-60]	0.550
Oto Score**		3 [2-4]	3 [2-4]	3 [1–5]	0.739
Cold Ischemia Time (min)		450 [370-664]	443 [378-603]	472 [349-785]	0.565
Warm Ischemia Time (min)		73 [66-85]	72 [64-88]	74 [67-84]	0.793
DCD		16 (19%)	7 (13%)	9 (30%)	0.067
EVLP		18 (21%)	9 (17%)	9 (30%)	0.159
Induction Therapy		71 (84%)	46 (85%)	25 (83%)	0.822
RBC (units)		4 [2-8]	3.5 [2.0-6.0]	5.5 [3.0-10.2]	**0.024**
FFP (units)		4 [2-6]	4.0 [0.0-6.0]	4.0 [2.0-8.0]	0.138
Blood Components (L)		2.6 [1.3-4.1]	2.6 [7.25-3.6]	3.1 [2.0-4.7]	**0.027**
Intraoperative ECMO (y/n)		45 (53%)	25 (46%)	20 (67%)	0.070

Data are presented as the absolute frequency (% of the included patients) or as median and interquartile range. AKI, Acute Kidney Injury; BMI, Body Mass Index; CF, Cystic Fibrosis; COPD, Chronic Obstructive Pulmonary Disease; CKD, Chronic Kidney Disease: defined as an eGFR CDK-EPI (2021 definition) <90 mL/min/1.73 m² ECMO, extracorporeal membrane oxygenation; DCD, Donor after Cardiac Death; EVLP, Ex-Vivo Lung Perfusion; RBC, Red Blood Cell; FFP, Fresh Frozen Plasma. **) not calculated for graft undergone EVLP.

Twelve (14%), 7 (8%), and 11 (13%) developed AKI stage 1, 2, and 3, respectively. One patient died before post-operative day 7, required RRT, and thus was considered to be suffering AKI stage 3.

In the overall cohort, sCr peaked at 3 [1-4] days after LUTX, at 0.99 [0.72-1.33] mg/dL. Among AKI patients, peak sCr occurred at 4 [3-5] days, at 1.60 [1.18-2.00] mg/dL. Using sCr criteria alone, AKI was diagnosed in 28 patients (93% of AKI cases), with the earliest diagnosis at 2 [1-3] days after LUTX. Oligo-anuria (i.e., UO < 0.3 mL/kg/24 hours) occurred in 7 (8%) patients, at 3 [1-5] days after LUTX, and only in 2 patients was UO the sole diagnostic criterion. Seven patients (8.3%) needed RRT during the ICU stay, and 3 additional patients required RRT after ICU discharge. RRT was commenced 6 [3-15] days after LUTX and lasted 9 [3-26] days.

Table S1 (Online Supplement, Additional Results) depicts the impact of AKI on outcomes. At 90 days, 6 patients had died. AKD occurred in 29 (97%) of AKI patients, as compared to 33 (62%) of patients without AKI (*p* < 0.001) (see Figure S2, Online Supplement, Additional Results), while CKD at 1 year was similarly very frequent, with CKD ≥3 occurring in 32 (59)% vs 22 (73%) in AKI = 0 and AKI>0 cohorts, *p* = 0.192). AKI was associated with higher PGD incidence (see Figure S2, Online Supplement, Additional Results), fewer ventilation-free, ICU-free, and hospital-free days (all *p* < 0.001), and increased mortality (log-rank *p* = 0.007) (see Figure S3, Online Supplement, Additional Results).

### Urinary [TIMP-2]*[IGFBP-7] as a predictor of AKI

**Figure 1:**
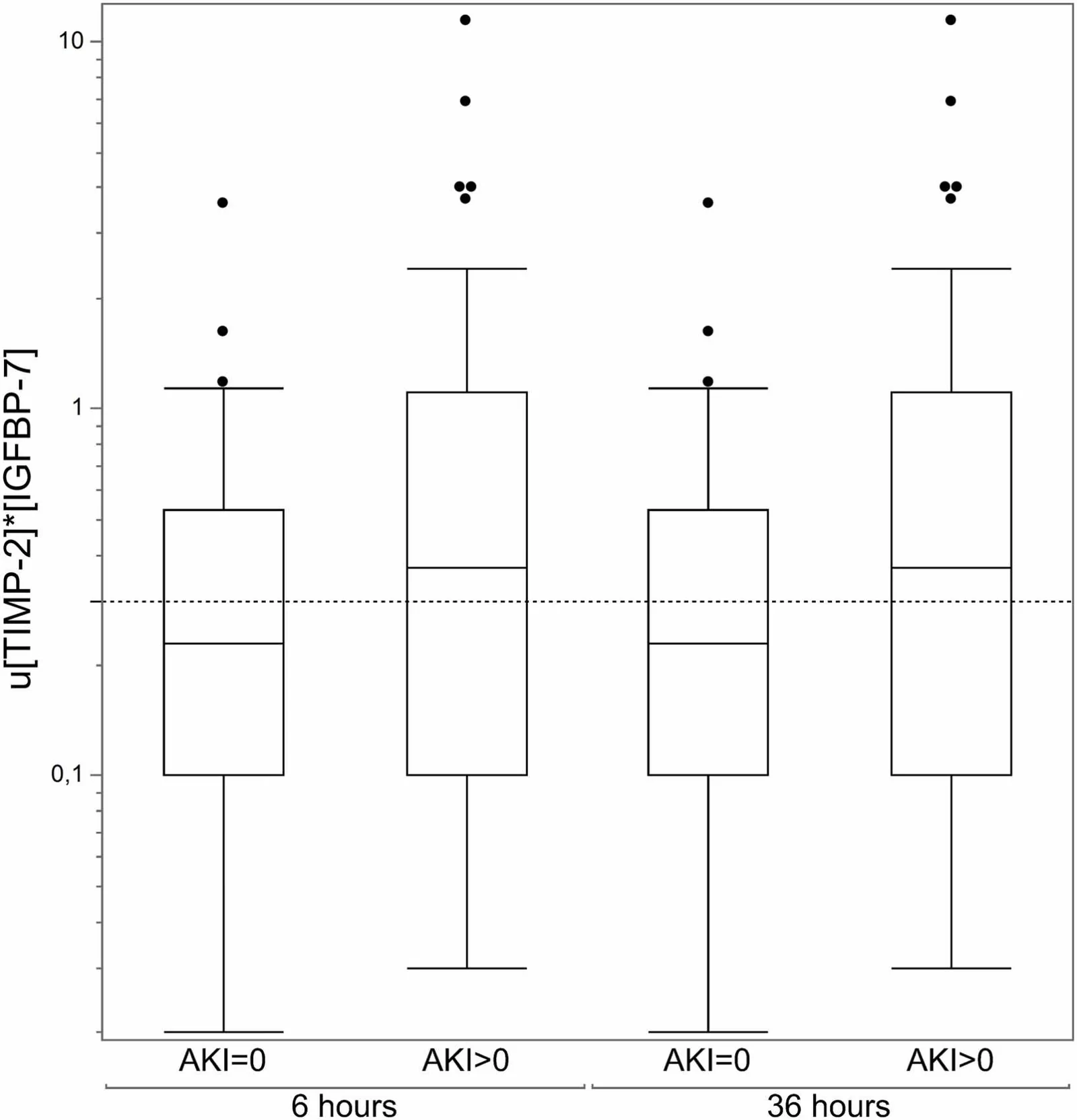
Urinary [TIMP-2]*[IGFBP-7] concentrations at 6 and 36 hours according to Acute Kidney Injury (AKI) status. Box plots show u[TIMP-2]*[IGFBP-7] measured at 6 and 36 hours after reperfusion, stratified by AKI status. The y-axis is displayed on a logarithmic scale. The horizontal dashed line represents the 0.3 cutoff for u[TIMP-2]*[IGFBP-7]. TIMP-2, Tissue Inhibitor Of Metalloproteinases-2; IGFBP-7, Insulin-Like Growth Factor Binding Protein-7.

Figure 1 depicts u[TIMP-2]*[IGFBP-7] across timepoints, stratified by AKI status. At ICU admission, the median u[TIMP-2]*[IGFBP-7] was 0.155 [0.070-0.480] (ng/mL)^2^/1000, and did not differ according to AKI status (*p* = 0.400). At 36 hours, the median u[TIMP-2]*[IGFBP-7] was 0.275 [0.100-0.675] (ng/mL)^2^/1,000, with higher values in AKI patients (*p* = 0.008, OR per unit increase 1.73 [1.11-3.47]).

Six hours after graft reperfusion, u[TIMP-2]*[IGFBP-7] was > 0.3 in 25 patients (29%), among whom 13 (52%) developed AKI, compared with 18 of 59 patients (30%) with values ≤0.3 (*p* = 0.062, OR 2.48 [0.96-6.58]). At the same time point, 5 patients already met the KIDGO criteria for AKI. At 36 hours, u[TIMP-2]*[IGFBP-7] was > 0.3 in 39 patients (48%), among whom 19 (57%) developed AKI, compared with 14 of 41 patients (42%) with values ≤0.3 (*p* = 0.118; OR 1.80 [0.74-4.57]). Fourteen patients met the KDIGO criteria for AKI at 36 hours. Discriminatory performance of u[TIMP-2]*[IGFBP-7] >0.3 in predicting AKI was modest (Table S2 and Figure S4, Online Supplement, Additional Results), with AUROC 0.56 (95%CI 0.48, 0.63) at 6 hours and 0.63 (95%CI 0.58, 0.70) at 36 hours after graft reperfusion.

### Subclinical AKI defined by [TIMP-2]*[IGFBP-7]

Among the 53 patients without KDIGO-defined AKI, 25 (47%) had u[TIMP-2]*[IGFBP-7] >0.3 at either 6 or 36 hours after graft reperfusion and were classified as having subclinical AKI. No statistically significant difference in the worst ICU CrCl was documented between patients with AKI=0 (biomarker negative) and subclinical AKI (i.e., 103 [68-128] vs 100 [65-119] mL/min, p=0.678; mean difference −5 mL/min (95%CI −34, 16), while AKI>0 had lower worst CrCl than AKI=0 and subclinical AKI patients cohorts (*p* < 0.001, for both).

Subclinical AKI was not associated with subsequent AKD (*p* = 0.281, OR 0.53 [0.16-1.66]). Compared with patients without AKI and u[TIMP-2]*[IGFBP-7] ≤0.3, subclinical AKI was not associated with a statistically significant differences in 28 days ventilator-free days (difference −2.8 days (95%CI −6.2, 0.5); *p* = 0.099), ICU-free days (difference −2.0 days (95%CI −4.7, 0.1); *p* = 0.136), but with a reduction in 28 days organ support-free days (difference −3.9 days (95%CI −7.8, 0.7); *p* = 0.042), (see Table S3, Online Supplement, Additional Results). No statistically significant difference between subclinical AKI and AKI = 0 was detected in 6-month rejection (*p* = 0.987, in AKI = 0 26% vs subclinical AKI 25%), CKD ≥3 (*p* = 0.645, in AKI = 0 65% vs 56% in subclinical-AKI), or survival at follow-up (log-rank *p* = 0.479, in AKI = 0 80% vs 88% subclinical-AKI).

### Plasma biomarkers as a predictor of AKI

**Figure 2:**
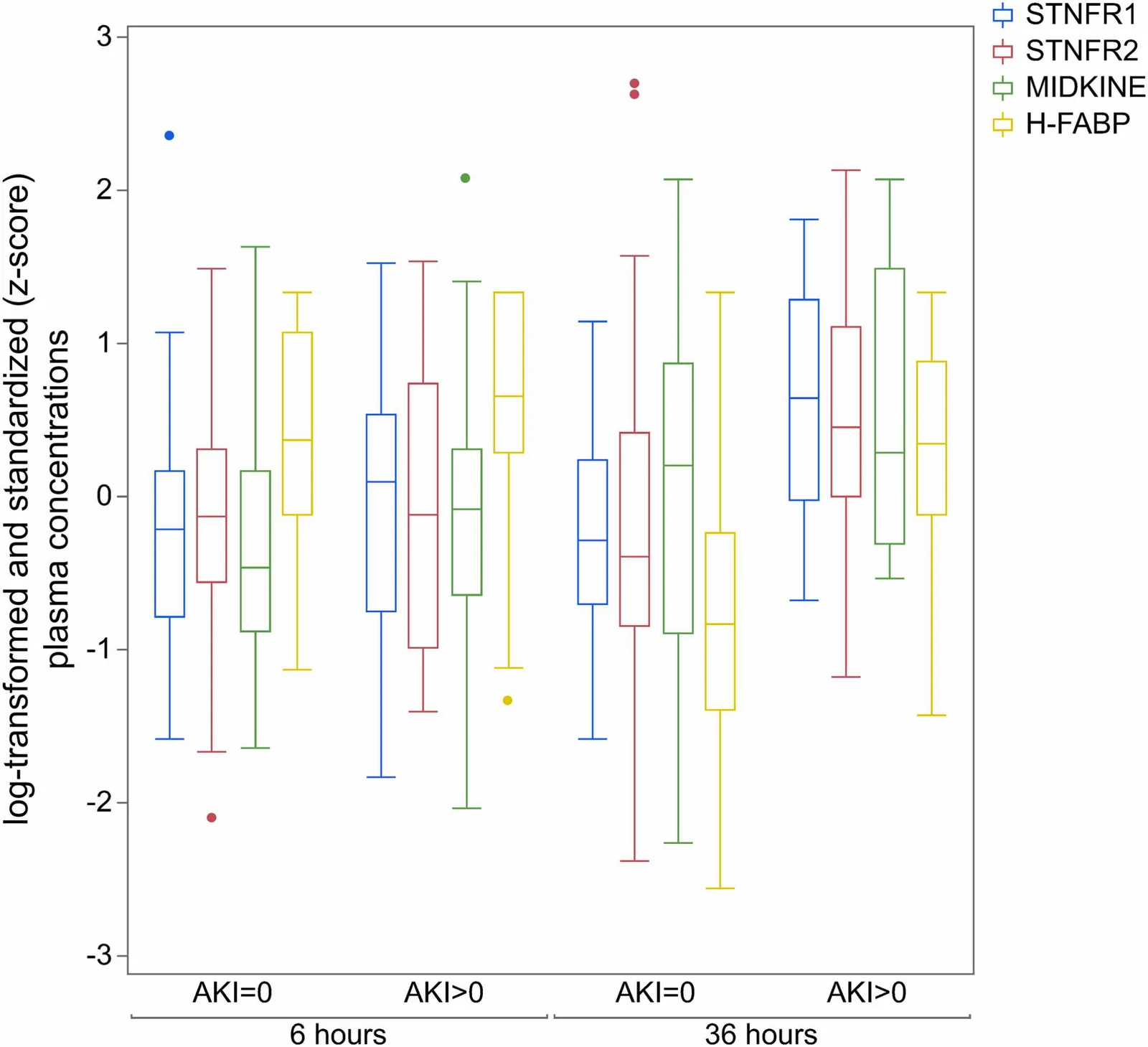
*Biomarkers plasma concentrations at 6 And 36 hours stratified by acute kidney injury (AKI) status.* Boxplots depict log-transformed and standardized (z-score) plasma concentrations measured at 6 and 36 hours after reperfusion, stratified by AKI status. sTNFR, Soluble Tumor Necrosis Factor Receptor; H-FABP, Heart-Type Fatty Acid–Binding Protein; AKI, Acute Kidney Injury.

Figure 2 and Table S4 (Online Supplement, Additional Results) depict H-FABP, midkine, sTNFR1, and sTNFR2 stratified by AKI status. At 6 hours, no statistically significant difference was detected in the biomarkers’ concentration according to AKI status. At 36 hours, patients with AKI>0 had statistically significant higher concentrations of H-FABP (*p* < 0.001), sTNFR1 (*p* = 0.002), and sTNFR2 (*p* = 0.004), but not midkine (*p* = 0.095). At 36 hours, the plasma biomarkers showed heterogeneous discriminatory performance, with AUROCs ranging from 0.59 (95%CI 0.52-0.67) for midkine to 0.82 (95%CI 0.79-0.87) for H-FABP. These estimates were numerically higher than those observed for urinary u[TIMP-2]*[IGFBP-7] Figure S5 (Online Supplement, Additional Results).

### LPA-derived composite biomarker as a predictor of AKI

Of the 84 included patients, complete biomarker data were available in 73, who were included in the LPA. At 6 hours, LPA did not identify a stable multi-class solution, with models yielding a dominant cluster and secondary groups with < 3 patients (see Table S5, Online Supplement, Additional Results). At 36 hours, the 3-class solution was selected as the preferred exploratory model according to the BIC, with complementary consideration of the Akaike Information Criterion, entropy, class size, and biological interpretability. (see Table S6, Online Supplement, Additional Results). The classes comprised 22 (30%), 37 (51%), and 14 (19%) patients, respectively, with an entropy of 0.804.

To allow prospective assignment of new patients to the predefined phenotypes, an exploratory individual-level phenotype classifier was derived directly from the final LPA model, without additional model fitting (see Online Supplement, Additional Results). For each patient, the classifier generated posterior probabilities of membership in the phenotypes and assigned the patient to the class with the highest posterior probability. The classifier uses the same four 36-hour biomarkers and the fixed preprocessing and model parameters derived from the study cohort.

**Figure 3:**
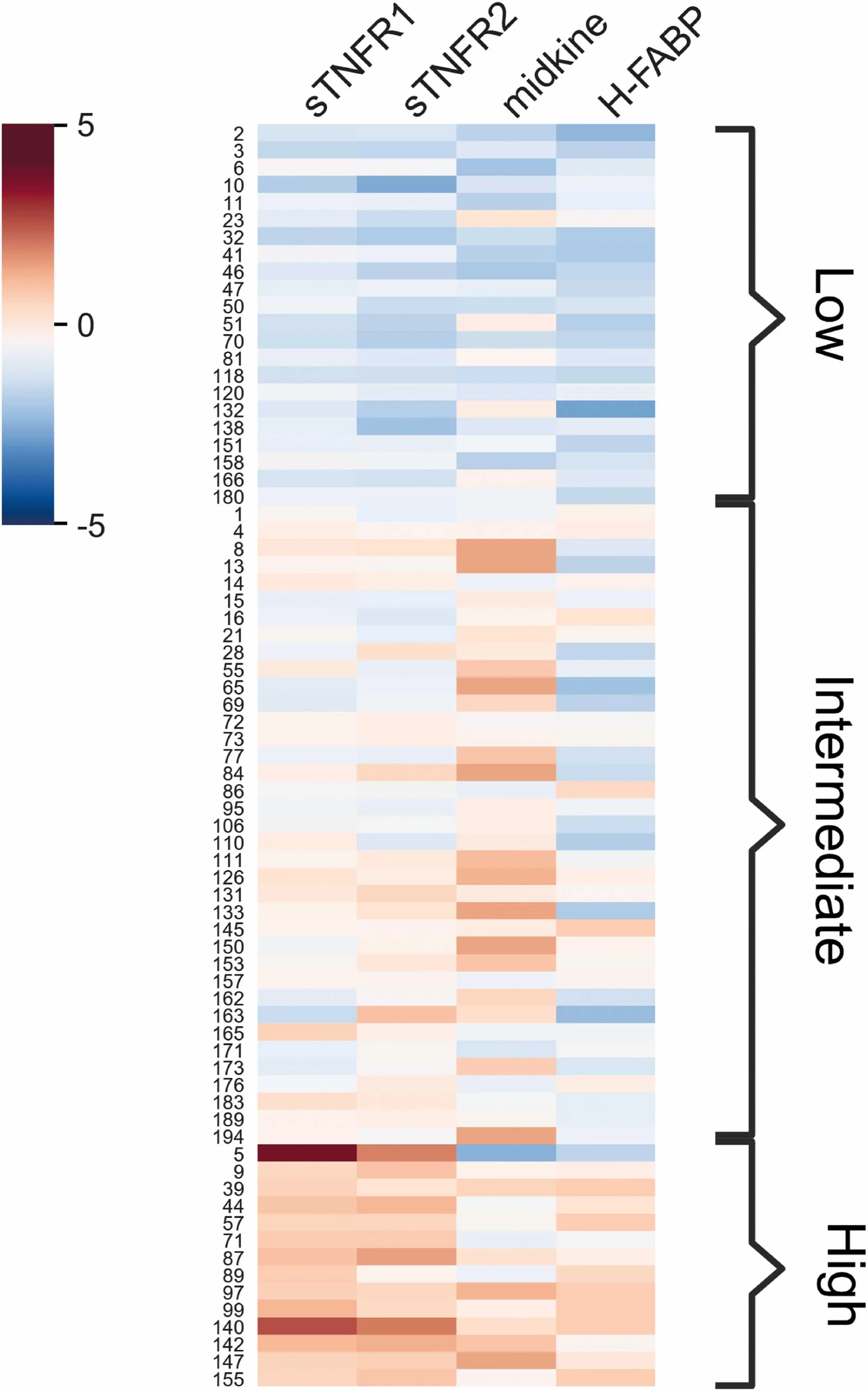
*Heatmap of plasma biomarker concentrations at 36 hours according to latent profile analysis.* Heatmap representation of log-transformed and standardized (z-score) plasma concentrations of sTNFR1, sTNFR2, midkine, and H-FABP measured 36 hours after graft reperfusion in patients with complete biomarker data (*n* = 73). Each row represents an individual patient, ordered by LPA-derived class membership (low, intermediate, and high). Colors indicate relative biomarker expression (blue = lower than cohort mean; red = higher than cohort mean). sTNFR, Soluble Tumor Necrosis Factor Receptor; H-FABP, Heart-Type Fatty Acid–Binding Protein.

Biomarker concentrations in the 3 classes are shown in Table 2 and Figure 3. A stepwise increase was observed for sTNFR1 and sTNFR2 from the low- to the high-concentration biomarker class (both *p* < 0.001). H-FABP demonstrated a similar gradient, with the highest levels in the high-biomarker class. Midkine concentrations were higher in the intermediate- and high-biomarker classes compared with the low-risk class (*p* < 0.001).

**Table 2:** Biomarker Concentrations Stratified by Latent Profile Analysis Phenotypes at 36 Hours After Reperfusion

	Low-risk *N* = 22	Intermediate-risk *N* = 37	High-risk *N* = 14	*p*
sTNFR1 (ng/mL)	1.41 [1.17-1.59]	2.05 [1.64-2.29]^1^	3.57 [3.30-4.39]^1^, ^2^	<0.001
sTNFR2 (ng/mL)	0.25 [0.19-0.35]	0.52 [0.40-0.69]^1^	1.21 [0.97-1.77]^1^, ^2^	<0.001
midkine (ng/mL)	5.53 [3.64-9.55]	15.16 [10.47-32.61]^1^	13.11 [9.22-25.66]^1^	<0.001
H-FABP (ng/mL)	11.53 [10.08-17.08]	23.09 [13.29-31.35]^1^	50.36 [34.13-67.59]^1^, ^2^	<0.001

Plasma biomarker concentrations measured 36 hours after reperfusion according to the 3 phenotypes identified by latent profile analysis (low-, intermediate-, and high-risk). Data are reported as median [interquartile range]. *P* values refer to the overall comparison across classes. Superscript numbers indicate significant pairwise differences: 1 vs low class; 2 vs intermediate class. sTNFR1, Soluble Tumor Necrosis Factor Receptor 1; sTNFR2, Soluble Tumor Necrosis Factor Receptor 2; H-FABP, Heart-Type Fatty Acid-Binding Protein.

**Figure 4:**
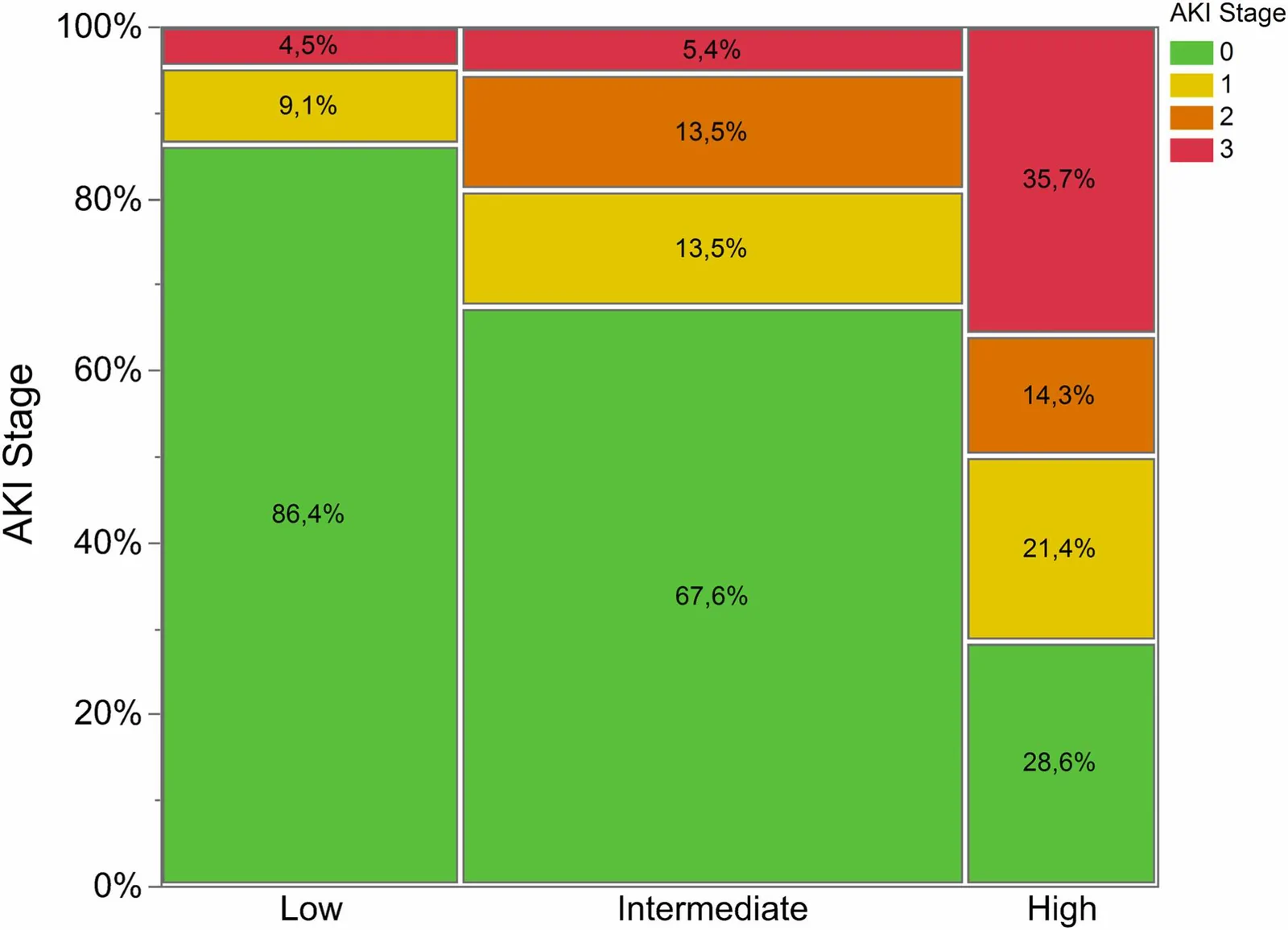
*Distribution of Acute Kidney Injury (AKI) stages across phenotypes at 36 hours after reperfusion.* Mosaic plot showing the distribution of Acute Kidney Injury (AKI) stages (0-3) across the 3 latent profile analysis phenotypes identified from plasma biomarker measurements obtained 36 hours after reperfusion (low, intermediate, and high). The width of each column is proportional to the number of patients in the corresponding phenotype, and the height of each colored segment represents the proportion of patients within each AKI stage. Percentages are reported within each class.

**Figure 5:**
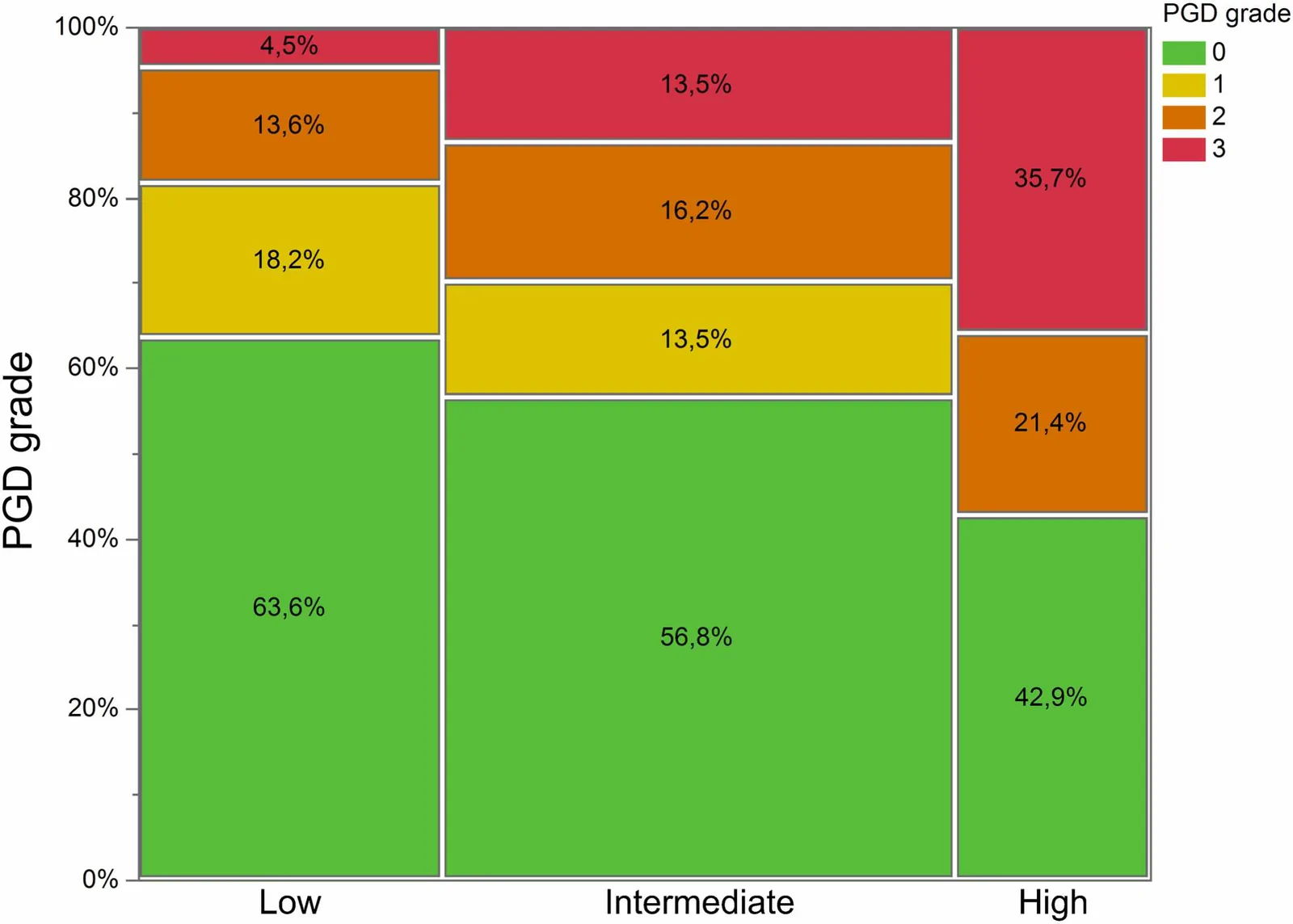
*Distribution of Primary Graft Dysfunction (PGD) grades across latent profile phenotypes at 36 hours after reperfusion.* Mosaic plot showing the distribution of primary graft dysfunction (PGD) stages (0-3) across the 3 latent profile analysis phenotypes identified from plasma biomarker measurements obtained 36 hours after reperfusion (low, intermediate, and high). The width of each column is proportional to the number of patients in the corresponding class, and the height of each colored segment represents the proportion of patients within each AKI stage. Percentages are reported within each class.

A graded distribution of postoperative AKI severity was observed across LPA classes (*p* = 0.006) (see Figure 4), with AKI occurring in 16% of the patients in the low-biomarker class up to 72% in the high-biomarker class, and accordingly, a progressive worsening of CrCl was observed among classes (*p* < 0.001) (see Table 3). Thus, the low-, intermediate- and high-biomarker classes were interpreted as kidney-injury classes and defined as low-risk, intermediate-risk, and high-risk phenotypes. We also observed a graded increase in the risk of PGD was observed among those kidney-injury phenotypes (*p* = 0.045), see Figure 5. Table 3 summarizes the impact on outcome stratified by phenotypes. The high-risk phenotype exhibited reduced organ-support–free days, ICU-free days, and hospital-free days compared with the intermediate and low-risk phenotypes. A trend towards higher incidence of AKD was observed for the high-risk class (*p* = 0.143, with OR 3.0 (0.58, 22.80) for AKD risk in the high- vs low-risk class). Exploratory post-hoc analyses documented a gradient of long-term compromise across classes. At 6 months, rejection-free survival decreased from 77% in the low-risk class to 70% in the intermediate-risk class and 42% in the high-risk class, alongside increasing proportions of rejection (18%, 22%, and 43%, respectively) and death (4%, 8%, and 14%, respectively) (p for trend = 0.043), with each increase in class associated with higher odds of adverse 6-month outcome (OR 2.12, 95%CI 1.01-4.46; *p* = 0.048) (see Figure S6, Online Supplement). Similarly, at 1 year, CKD categories differed across phenotypes (*p* = 0.036), with CKD category ≥3 more common in the intermediate- and high-risk cohorts (45%, 78%, and 71%, respectively, *p* = 0.034). With a median follow-up of 769 [520-1121] days, no statistically significant difference in survival was detected (log-rank *p* = 0.209) among risk phenotypes.

**Table 3:** Clinical Outcomes Stratified by Latent Profile Analyses Classes

	Low-risk *N* = 22	Intermediate-risk *N* = 37	High-risk *N* = 14	p*
PGD grade 3 (72 hours)	1 (4%)	5 (13%)	5 (35%)	0.045
IMV, days	1 [1-3]	1 [1-3]	5 [1-21]	0.125
28-Days IMV free days	27 [24-27]	27 [24-27]	23 [7-27]	0.094
28-days organ support free days	27 [23-27]	26 [24-27]	23 [0-26]	0.005
28-days ICU free days	24 [22-26]	24 [22-25]	16 [0-25]	0.009
CrCl (mL/min)	86 [62-128]	72 [39-108]	27 [4-57]	<0.001
AKD >0	14 (66%)	28 (75%)	12 (85%)	0.134
60-days Hospital Free Days	33 [25-42]	35 [4-40]	12 [0-34]	0.037
CKD ≥3 at 1 year	10 (45%)	29 (78%)	10 (71%)	0.034
Death at follow-up	3 (13%)	9 (24%)	5 (35%)	0.290**

Acute Kidney Injury; AKD, Acute Kidney Disease; PGD, Primary Graft Dysfunction; IMV, Invasive Mechanical Ventilation; ICU, Intensive Care Unit; CrCl, Creatinine Clearance; Chronic Kidney Disease, CKD. *) ordinal logistic. **) log-rank.

Patients' characteristics at baseline stratified by phenotypes are summarized in Table S7 (Online Supplement, Additional Results). Diabetes and pre-existing CKD were more prevalent in the high-risk phenotype (*p* = 0.045 and *p* = 0.027, respectively), whereas other clinical and perioperative variables did not differ significantly.

In sensitivity post-hoc exploratory analyses, aiming at correcting for baseline differences among phenotypes, the association between LPA phenotype and AKI stage remained significant after adjustment for u[TIMP-2]*[IGFBP-7] positivity (*p* < 0.001) and baseline CKD and diabetes (*p* = 0.031).

An exploratory individual-level phenotype classifier was derived directly from the final 3-class LPA model, without additional model fitting (see Online Supplement, Additional Results). For each patient, the classifier generated posterior probabilities of membership in the low-, intermediate-, and high-biomarker phenotypes and assigned the patient to the class with the highest posterior probability. The classifier uses the same four 36-hour biomarkers and the fixed preprocessing and model parameters derived from the study cohort and is intended to allow prospective assignment of new patients to the predefined phenotypes. The model is not for actual clinical use but is made available as an Online Digital Supplement, for future external validation.”

Sensitivity analysis aiming at testing clustering robustness by HCA identified a 3-cluster structure closely matching the LPA-derived phenotypes (see Online Supplement, Figure S7), near-perfect correlation between corresponding cluster-specific standardized biomarker profiles (*r* = 0.989, *p* < 0.001) (see Online Supplement, Table S8), with 86.3% concordance in patient classification, and highly concordant associations within HCA-derived clustering and the primary outcome AKI (see Online Supplement, Figure S8).

## Discussion

In this prospective observational cohort study of LUTX recipients, postoperative AKI was frequent, clinically impactful, and difficult to identify early with serum creatinine or UO. In this setting, u[TIMP-2]*[IGFBP-7] had modest predictive power for AKI, and subclinical AKI defined with positive u[TIMP-2]*[IGFBP-7] was not associated with a clearly distinct clinical course. In contrast, outcome-agnostic LPA of plasma H-FABP, midkine, sTNFR1, and sTNFR2 identified 3 creatinine-independent risk phenotypes with graded differences in AKI severity and postoperative trajectory.

Previous literature reported that AKI after LUTX occurs frequently (>50% patients) and is associated with prolonged organ support and reduced survival,^33^ findings consistent with our data. Since AKI treatment is just supportive, early risk stratification may allow predictive enrichment and targeted management approaches. In critically ill patients, several biomarkers have been evaluated for this scope,^16^ and subclinical AKI has been proposed as a new clinical entity characterized by biological evidence of tubular injury without overt functional impairment.^34^ At the time of this writing, however, there is only a single FDA-approved assay for this purpose, which is based upon the measurement of the urinary concentration of the cell-cycle arrest proteins TIMP-2 and IGFBP-7.^35^ These proteins are part of the tubular cell-cycle molecular machinery and are secreted in the urinary tract in response to several renal insults,^36^ resulting in transient interruption of tubular cell replication. The detection of these biomarkers in the UO is interpreted as impending tubular stress. The product of their urinary concentration (i.e., u[TIMP-2]*[IGFBP-7]) has been shown to predict AKI and stratify risk in several surgical cohorts,^37^, ^38^ comprising solid organ transplant recipients.^29^ The implementation of renal protective bundles guided by u[TIMP-2]*[IGFBP-7] reduced the incidence of moderate-to-severe AKI compared with standard care.^39^ Nevertheless, evidence supporting clinical implementation of u[TIMP-2]*[IGFBP-7] in LUTX is lacking, while newer biomarkers continue to be explored for AKI risk stratification.

With these premises, we sought to assess in LUTX recipients the predictive performance of the established urinary biomarker u[TIMP-2]*[IGFBP-7] and also of a novel creatinine-independent outcome-agnostic profiling strategy based on plasma concentration of H-FABP, midkine, sTNFR1, and sTNFR2.

In our cohort, u[TIMP-2]*[IGFBP-7] predicting capabilities were modest, and subclinical AKI defined with u[TIMP-2]*[IGFBP-7] was poorly associated with subsequent renal and overall clinical course. Several factors may account for these findings. First, perioperative management during LUTX (e.g., large intraoperative fluid administration, postoperative negative fluid-balance strategies) may reduce the diagnostic performance of urinary cell-cycle arrest biomarkers. Second, sampling time may be relevant, as tubular stress detectable by u[TIMP-2]*[IGFBP-7] may emerge later in the postoperative course. Third, hemodynamic perturbation, ischemia-reperfusion, and inflammatory activation may generate an early circulating injury signal that is more readily captured in plasma than in urine, whereas tubular stress detectable by u[TIMP-2]*[IGFBP-7] may be delayed, attenuated, or not central in this cohort.

In this preliminary analysis, a plasma-based approach comprising H-FABP, midkine, sTNFR1, and sTNFR2, combined with outcome-agnostic LPA, identified 3 biomarker-defined phenotypes at 36 hours after graft reperfusion. The 3 phenotypes were associated with a graded pattern of postoperative risk. The low-risk phenotype comprised about 30% of patients and was characterized by infrequent AKI, less PGD, and a benign postoperative course. At the other end of the spectrum, the high-risk phenotype accounted for about 20% of the cohort and showed frequent AKI, greater severity of kidney injury, lower AKD-free survival, more PGD, and a more complex postoperative course with prolonged need for organ support and longer ICU stay. The intermediate-risk phenotype, representing about half of the population, showed intermediate outcomes.

H-FABP, midkine, sTNFR1, and sTNFR2 are not specific to kidney injury or damage and capture different aspects of the perioperative LUTX course. H-FABP is a myocardial cytosolic protein^40^ and its elevation may derive from perioperative myocardial injury. Midkine is a pleiotropic heparin-binding cytokine with anti-apoptotic, pro-angiogenic, and pro-inflammatory functions,^41^ its plasma concentration elevation likely reflecting IRI. sTNFR1 and sTNFR2 are soluble markers of TNF-pathway activation and represent markers of systemic pro-inflammatory pathway activation. Taken together, these biomarkers may be viewed as components of an integrated systemic response to the perioperative burden of LUTX. These pathways are biologically interconnected: myocardial and right ventricular dysfunction may reduce effective renal perfusion, ischemia-reperfusion and endothelial injury may amplify microvascular and tubular stress, and persistent systemic inflammation may further promote endothelial dysfunction and multiorgan injury. Within this framework, the LPA phenotypes may represent different systemic biological responses to the transplant-related insult, characterized by varying degrees of cardiocirculatory stress, ischemia-reperfusion injury, endothelial activation, and inflammation, rather than sequential stages of kidney injury alone. This interpretation is consistent with the parallel associations observed with AKI and PGD. Indeed, these phenotypes stratified not only AKI severity but also the overall postoperative trajectory. In particular, the parallel association of AKI and PGD supports the hypothesis that AKI and PGD may share systemic pathophysiology. IRI leading to endothelial dysfunction, complement activation, and neutrophil recruitment may therefore affect both the pulmonary microcirculation of the transplanted graft and the renal microvasculature, promoting pulmonary capillary leak and graft dysfunction while reducing renal perfusion and amplifying tubular and microvascular injury. Of note, we observed that divergence in biomarker-defined phenotypes was not observable in the immediate post-operative period but occurred over time. This is consistent with our overall interpretation of phenotypes of risk and may indicate that the relevant biological response develops progressively after transplantation, as sustained inflammation, endothelial dysfunction, hemodynamic perturbation, and evolving multiorgan injury become established.

The phenotypic signal persisted after adjustment for baseline characteristics associated with AKI (i.e., CKD and diabetes) and was independent of u[TIMP-2]*[IGFBP-7] and serum creatinine as well. In this framework, H-FABP, midkine, sTNFR1, and sTNFR2 are best interpreted not as instantaneous markers of kidney-specific structural injury, but as complementary indicators of evolving cardiorenal, ischemia-reperfusion, and inflammatory responses. Considered jointly through LPA, these biomarkers revealed clinically meaningful heterogeneity in early post-transplant kidney injury that was not captured by single-marker strategies. However, the present study does not establish their clinical utility *per se* as diagnostic tests or for routine patient management.

From a clinical perspective, biological phenotyping could be used to identify patients who may benefit from intensified surveillance. Indeed, in a future validated pathway, assignment to a high-risk phenotype could prompt closer monitoring and consideration of a kidney-protective care bundle, comprising hemodynamic optimization, nephrotoxin exposure limitations, and tighter calcineurin-inhibitor monitoring. More importantly, these findings provide a rationale for moving beyond current syndromic approaches to AKI prevention and for future studies testing whether early biological phenotyping may help identify and target the predominant pathophysiological pathways of AKI. To allow future studies aimed at validating this approach in more populated multicenter studies, an individual-patient classifier model was developed and made available online.

However, whether biological profiling improves prediction beyond established clinical risk factors remains unresolved. Patients undergoing LUTX may have several relevant determinants of postoperative AKI, including baseline renal function, diabetes, perioperative ECMO support, hemodynamic instability, and transfusion exposure. The present study did not aim at and was not powered to construct a comprehensive clinical prediction model including these variables and to compare it formally with a model incorporating the biomarker phenotype. Furthermore, populated studies are necessary to confirm the clinical applicability of this approach.

Exploratory post hoc analyses also suggested a gradient in 6-month clinical outcome across phenotypes, with the high-risk class showing the lowest rejection-free survival and the highest proportion of adverse outcomes, and CKD, with the low-risk class showing the lowest risk of CKD, although longer-term survival did not differ significantly. Still, these associations should be considered exploratory and hypothesis-generating, given the small sample size and limited number of long-term events.

Several limitations should be acknowledged. First, this was a single-center study with a relatively small cohort, which may limit generalizability. Second, the population lacked substantial ethnic diversity. Third, the plasma biomarker phenotypes were derived and evaluated within the same dataset, in a relatively small complete-case cohort. External validation in independent multicenter cohorts is required. Fourth, the study was not powered to construct and validate a comprehensive clinical prediction model including all relevant perioperative risk factors. Larger prospective multicenter studies are needed for this scope.

In conclusion, plasma biomarker-derived phenotypes identified 36 hours were associated with graded differences in AKI severity and postoperative trajectory after LUTX, whereas u[TIMP-2]*[IGFBP-7] had limited early discriminatory performance. These results suggest that integrated plasma biomarker profiling may reveal clinically meaningful biological heterogeneity in early post-transplant kidney injury. Further studies in larger and independent cohorts are warranted to validate this approach and explore its role in risk stratification and mechanistic understanding of AKI after LUTX.

## Authors' Contributions

VS conceived the study; VS, AU, GT, SMC, MB, VV, DC, MB collected clinical, respiratory function data and data regarding pre, intra, and post-operative management; LMC performed patients' follow-up and collected follow-up data; VS, performed data analyses; VS, drafted the original version of the manuscript; SMC, GC, AZ, MN, LR, FB, and GG revised the manuscript critically; all authors approved the final version of the manuscript.

## Disclosure statement

Vittorio Scaravilli has received congress support from Biomerieux Italia S.p.a. (Bagno di Ripoli, Firenze, Italy) and Randox (Crumlin, UK) and serves as scientific consultant for U-Care Medical S.r.l. (Turin, Italy). Sebastiano Maria Colombo has received congress support from AOP Health (Pisa, Italy) and from BD Italia (Milano, Italy). Valentina Vago has received congress support from Biomerieux (Bagno di Ripoli, Firenze, Italy). Francesco Blasi reports receipts of grants from AstraZeneca, GSK, and Insmed; personal consulting fees from Menarini; personal fees for lectures, presentations, speakers bureaus, or educational events from AstraZeneca, Boehringer Ingelheim, Chiesi, GSK, Grifols, Insmed, Menarini, OM Pharma, Pfizer, Vertex, and Zambon, outside the submitted work. Giacomo Grasselli has received payments for scientific presentations from Dräger Medical, Getinge, Fisher & Paykel, Mundipharma, and Cook Medical; participated in advisory board activities for GSK; and has received research grants from Fisher & Paykel and MSD. The remaining authors do not have conflict of interest or financial disclosures.

## Financial support

The study was (partially) supported by a Ricerca Corrente funds, the Italian Ministry of Health, and Linea 2 Funds (G43C24002040001), Department of Biomedical, Surgical, and Dental Sciences, University of Milan.

## Conflict of Interest statement

The authors declare the following financial interests/personal relationships, which may be considered as potential competing interests: Vittorio Scaravilli has received congress support from Biomerieux Italia S.p.a. (Bagno di Ripoli, Firenze, Italy) and Randox (Crumlin, UK) and serves as scientific consultant for U-Care Medical S.r.l. (Turin, Italy). Sebastiano Maria Colombo has received congress support from AOP Health (Pisa, Italy) and from BD Italia (Milano, Italy). Valentina Vago has received congress support from Biomerieux (Bagno di Ripoli, Firenze, Italy). Francesco Blasi reports receipts of grants from AstraZeneca, GSK, and Insmed; personal consulting fees from Menarini; personal fees for lectures, presentations, speakers bureaus, or educational events from AstraZeneca, Boehringer Ingelheim, Chiesi, GSK, Grifols, Insmed, Menarini, OM Pharma, Pfizer, Vertex, and Zambon, outside the submitted work. Giacomo Grasselli has received payments for scientific presentations from Dräger Medical, Getinge, Fisher & Paykel, Mundipharma, and Cook Medical; participated in advisory board activities for GSK; and have received research grants from Fisher & Paykel and MSD. The remaining authors do not have conflict of interest or financial disclosures.

## Footnotes

Appendix A Supplementary data associated with this article can be found in the online version at doi:10.1016/j.jhlto.2026.100679.
