# Supplementary material for: Early biomarkers of acute kidney injury after lung transplantation
Source: JHLT Open. 2026 Sep 2;14:100679. doi: 10.1016/j.jhlto.2026.100679 (PMC13583717; doi:10.1016/j.jhlto.2026.100679)
Supplement: Supplementary file 1 [file mmc1.docx]

**Online Supplement**

**Title**

Early Biomarkers of Acute Kidney Injury After Lung Transplantation

**Additional Methods**

**Lung Transplantation Management**

*LUTX Team*

Lung transplantation (LUTX) team at Fondazione IRCCS Ca' Granda - Ospedale Maggiore Policlinico is composed of two thoracic surgeons, one thoracic surgery fellows, a cardiac surgeon, two certified anesthesiologists, one thoracic anesthesia fellow, and one anesthesia fellow, a perfusionist, an operatory-room nurse and one surgical-nurse.

*LUTX Anesthetic preparation*

After routine monitor as per ASA guidelines, induction of general anesthesia with fentanyl (1-2 mcg kg^-1^), midazolam (1-2 mg) and propofol (1-3 mg kg^-1^), and muscle paralysis with rocuronium (0.6 mg kg^-1^) is achieved. Patients are intubated with large size single-lumen tube and undergo aggressive bronchoscopic toilette. Then, the single-lumen tube is substituted, and lung isolation is achieved with appropriately sized left-sided double-lumen endotracheal tube under bronchoscopic guidance. General balanced anesthesia is maintained with fentanyl (1-2 mcg kg^-1^ hr^-1^) and sevoflurane (end-tidal concentration 0.5%-1.0%), and muscle paralysis with sequential rocuronium boluses (0.15 mg kg^-1^). Patients are monitored with invasive right radial artery cannulation, central venous catheterization of the right internal jugular vein, and oximetric pulmonary artery catheterization capable of cardiac output monitoring by means of thermodilution technique. Trans-esophageal echocardiography is implemented pending the anesthesiologist preferences. A large bore (i.e., 7 Fr) catheter is introduced in the right antecubital vein and connected to a custom made rapid infusion system, capable of the infusion of up to 500 mL min^-1^. Throughout the procedure, patients are mechanically ventilated in volume control mode, with FiO_2_ to maintain SpO_2_> 90%, and minute ventilation to limit hypercapnia and acidosis.

Antibiotic prophylaxis is provided following the indication of infectious disease specialists and pre-operative airways cultures.

*LUTX Surgical procedure*

Patients are treated with sequential bilateral LUTX, using bilateral anterolateral thoracotomy with a transverse sternotomy or two anterior thoracotomies, pending the anatomical characteristics of the patients. After lysis of pleural and mediastinal adhesions, the vascular structures and bronchi are isolated. Then the less perfused lung (as per pulmonary perfusion scintigraphy) is disconnected from the mechanical ventilation and allowed to deflate. The pulmonary artery is cross-clamped, and the first cross-clamping 10 minutes test is performed: the surgical procedure is halted, hemodynamics are strictly monitored,and blood gas analyses are obtained every 2 minutes.

The veins, artery, andbronchus are sequentially resected. The graft is positioned in the thoracic cavity, and the bronchial, arterial and venous anastomoses are created. The lung is thoroughly de-aired before vascular unclamping to avoid systemic air emboli; the first lung graft is slowly re-perfused and connected to another mechanical ventilator. The graft is initially ventilated in pressure control mode with FiO_2_ 21%, PEEP of 10 cmH_2_O, RR of 4 bpm and plateau pressure of 25 cmH_2_O. A recruitment maneuver is applied to obtain complete lung inflation. Progressively, ventilation and oxygenation of the graft are increased to allow contralateral lung separation from ventilation. Particular attention is paid in limiting 1) FiO_2_ (i.e., < 50%); 2) driving pressures (i.e., < 15 cmH2O) and 3) de-recruitment (i.e., PEEP > 10 cmH_2_O) of the implanted graft. Then, contralateral native lung ventilation is interrupted, and the second pulmonary artery cross-clamping test is performed. Pneumonectomy of the second native lung and implantation of the second graft follows the procedure above. Finally, hemostasis is achieved, bilateral pleural drainage positioned, the thorax closed and the patient is transferred to the intensive care unit for follow-up.

*ECMO indication and pulmonary artery cross-clamping test.*

The pulmonary artery cross-clamping test is performed to simulate the hemodynamic conditions occurring during pneumonectomy in a controlled -and reversible- fashion, allowing the anesthesiologist to optimize hemodynamics and ventilation and eventually the cardiac surgeon to implement central veno-arterial ECMO in a semi-elective condition. During the first pulmonary artery cross-clamping test (while the native lung is ventilated and perfused), whether 1) pulmonary hypertension (i.e., systolic pulmonary artery pressure > 80 mmHg, 2) increase in PAPs > 50 mmHg associated with systemic hypotension (i.e., systolic arterial pressure < 60 mmHg) resistant to inotropic support; 3) major cardiac arrhythmias; 4) hypoxia (i.e., PaO_2_< 8 kPa, despite increasing FiO_2_ up to 100% and optimizing PEEP); 5) respiratory acidosis (i.e., pH < 7.25 despite increasing minute ventilation) the test is interrupted and ECMO implemented.

During the second pulmonary-artery cross-clamping (while the graft is ventilated and perfused), our policy is more protective towards hyperoxia and ventilator-induced lung injury. Thus, during the second test FiO_2_ is not increased > 50% and driving pressure is not increased> 15 cmH_2_O. If one of the mentioned conditions occur, ECMO is implemented.

During all the surgical procedure, intractable hypoxemia, acidosis, and hemodynamic failure may occur at any given moment, but 1) single lung ventilation, 2) cross-clamping tests and 3) reperfusion of the grafts are the most critical. Whether during the procure 1) pulmonary hypertension (i.e., systolic pulmonary artery pressure > 80 mmHg, 2) increase in PAPs > 50 mmHg associated with systemic hypotension (i.e., systolic arterial pressure < 60 mmHg) resistant to inotropic support; 3) major cardiac arrhythmias; 4) hypoxia (i.e., PaO_2_< 8 kPa, despite increasing FiO_2_ up to 100% to the native lung or 50% to the grafts and optimizing PEEP); 5) respiratory acidosis (i.e., pH < 7.25 despite increasing minute ventilation, while guaranteed driving pressure to the grafts < 15 cmH_2_O); the procedure is briefly halted, and ECMO implemented.

Our approach to intraoperative extracorporeal life support consists of central veno-arterial ECMO. After providing unfractionated heparin (i.e., 5000 UI) and eventual further boluses to achieve an aPTT>40 seconds, the ascending aorta and right atrium are cannulated. Blood is drained via a centrifugal pump directly to a polypropylene membrane lung where blood is oxygenated, decarboxylated, warmed and then directed to the central venous circulation. Initially, blood flow is set to achieve around 50% of the patient's cardiac output, gas flow to maintain normocapnia and fraction of oxygen in the sweep gas flow to maintain SpO_2_>95%. The extracorporeal circuit setting is dynamically modified during the procedure, pending the different surgical and anesthetic requirements and mean arterial pressure is maintained > 60 mmHg by increases in extracorporeal blood flow, but complete blood drainage and emptying of the heart is avoided, and the opening of the aortic valve is always guaranteed.

At the end of the surgical procedure, prior to chest closure, patients undergoing VA-ECMO undergo a progressive de-escalation of extracorporeal support, consisting of: 1) reduction of blood flows down to 1 L/min; 2) reduction of extracorporeal FiO_2_ down to 21%. Hemodynamics are monitored and blood gas analysis are collected serially, to verify the patient do not suffer from: 1) hemodynamic failure/right ventricle failure; 2) hypoxemia; 3) acidosis and hypercapnia and/or need to ventilate the patients with tidal volume > 6 mL kg^-1^ or driving pressure > 14 cmH2O. Whether hemodynamic failure is observed, central VA-ECMO is converted to peripheral VA support (i.e., femoral cannulations). Whether hypoxemia and/or hypercapnia is observed, VV-ECMO (i.e., femoral-femoral cannulation) is instituted and prolonged in the post-operatory period.

No predefined standard management of blood components is applied, but patient-tailored transfusion management is carried out following blood gas analyses, point of care (POC) PT/aPTT tests, and thromboelastography, as per national guidelines.

*Immunosuppressive therapy*

All recipients received induction therapy corticosteroids (methylprednisolone 1000 mg) at graft reperfusion. Moreover, patients received basiliximab (20 mg intravenously on postoperative days 0 and 4), except in the case of a previous history of neoplastic disease. Maintenance immunosuppression consisted of a triple-drug regimen including a calcineurin inhibitor (tacrolimus, Prograf®, oral formulation, twice a day), an antiproliferative agent (i.e., azathioprine), and corticosteroids. Tacrolimus drug dosing and serum level monitoring followed standard postoperative transplant practice (i.e., tacrolimus through level: 10-15 ηg mL^-1^ first 3 months, 8-12 ηg mL^-1^ up to 6 months).

*Post-operative Management*

We follow generally accepted guidelines for the post-operative management of LUTX recipients.

Protective mechanical ventilation is guaranteed. We limit tidal volume to 6 mL kg^-1^ of donor-predicted body weight and plateau pressure < 30 cmH_2_O. We use the lowest inspired oxygen fraction capable of providing an arterial oxygen saturation > 90%. Positive end-expiratory pressure (PEEP) is set following a decremental PEEP trial, with the usual initial PEEP being >= 10 cmH2O. Higher levels of PEEP are utilized in case of plasmorrhea and/or respiratory failure. Possible step-up therapies employed in case of primary graft dysfunction are prolonging sedation, introducing neuromuscular blockade, and ECMO, as previously mentioned. We pursue early weaning from mechanical ventilation, and thus the standard non-complicated patient is usually rapidly weaned from mechanical ventilation and extubated in the first 24 hours after graft reperfusion employing elective noninvasive ventilation. A dedicated respiratory therapist is available 12 hours/24 hours, 7 days/7 days to tailor the management of noninvasive support.

Hemodynamic management is guided by lactate, urinary output, invasive cardiac output monitoring, pulmonary artery pressure, wedge pressure, and mixed venous saturation measurement through an elective pulmonary artery catheter. We do not employ a specific hemodynamic protocol but follow the following general rules of thumb: a restrictive fluid strategy to maintain euvolemia, high-threshold red blood cell transfusions (i.e., > 9 gr L^-1^), and vasopressors are used to guarantee a mean arterial pressure in the 65-75 mmHg range and cardiac index (i.e., 2.2-2.5 L min^-1^ m^-2^).

Antimicrobial therapy is tailored to patients' and donors' characteristics. All patients receive prophylaxis for opportunistic infections: fungal (i.e., voriconazole), pneumocystis jirovecii (i.e., trimethoprim/sulfamethoxazole), and viral (i.e., ganciclovir). All patients receive surgical site infection prophylaxis for G- strains (i.e., cefepime for 72 hours) and G+ strains (i.e., vancomycin, one-shot). Cefepime is interrupted at 72 hours whether the donors' respiratory cultures are negative. Colonized patients continue their antibiotic treatment throughout the perioperative period. Strict infection surveillance is carried out during the post-operative period, by means of deep respiratory cultures collected at 24 hours, 5 days, and 15 days after LUTX and respiratory, anal, and perineal swabs for multi-drug resistant bacteria.

**Oto Score calculation**

A donor lung score was calculated as previously shown by Oto et al. (see Oto, Annals of Thoracic Surgery, 2007). For each donor, a score was obtained following the sum of each domain. For donors after cardiac death (n=4), the Oto score cannot be calculated.

| *Category* | *Stratification* | *Score* |
| --- | --- | --- |
| Age (years) | < 45 | 0 |
|  | 45-54 | 1 |
|  | 55-59 | 2 |
|  | ≥ 60 | 3 |
| Smoking history  (pack-years) | < 20 | 0 |
|  | 20-39 | 1 |
|  | 40-59 | 2 |
|  | ≥ 60 | 3 |
| Chest X-ray | Clear | 0 |
|  | Minor | 1 |
|  | Opacity ≤ 1 lobe | 2 |
|  | Opacity > 1 lobe | 3 |
| Secretions | None | 0 |
|  | Minor | 1 |
|  | Moderate | 2 |
|  | Major | 3 |
| PaO_2_/FIO_2_ | > 450 | 0 |
|  | 351-450 | 2 |
|  | 301-350 | 4 |
|  | ≤ 300 | 6 |

**Primary Graft Dysfunction Grading**

Primary graft dysfunction was evaluated following the latest ISHLT consensus (see Snell, Journal of Heart and Lung Transplant, 2017) at 72 hours from reperfusion, and graded as follows:

| *Grade* | *Radiographic Sign* | *PaO_2_/FIO_2_* |
| --- | --- | --- |
| 0 | No | > 300 |
| 1 | Diffuse pulmonary edema | > 300 |
| 2 | Diffuse pulmonary edema | 200 to 300 |
| 3 | Diffuse pulmonary edema | < 200 |

**AKI, AKD and CKD definition**

Serum Creatinine (sCr) was measured and collected at the following timepoints: enlistment, before LUTX (i.e., 6 hours before surgery commencement); each day during the ICU and hospital stay; at any ambulatory follow-up within 90 days of LUTX. Notably, the sCr measured 6 hours before surgery was considered baseline and utilized for diagnosis of renal dysfunction. At the same timepoints, the need for RRT was assessed. UO was assessed daily during ICU and hospital stay, up to the 7th post-operative day.

According to KDIGO criteria for AKI and ADQI16 workgroup classification for AKD patients were classified as having: a) post-operative AKI whether sCr increased ≥ 0.3 mg dL^-1^ at 48 hours, or in the first 7 days after LUTX sCr increased >1.5 times compared to pre-operative (stage 1, stage 2, and stage 3 whether sCr increased 1.5-1.9, 2-2.9, and >3 times, respectively) or having a UO <0.5 mL kg^-1^ hr^-1^ for 6-12 hours, < 0.5 mL kg^-1^ hr^-1^ for >12 hours, <0.3 mL kg^-1^ hr^-1^ for ≥24 hours or anuria for ≥12 hours; b) post-operative AKD whether sCr increased 1.5-1.9, 2-2.9, and >3 times (or need for dialysis) between 7 and 90 days after LT.

Long-term renal function was assessed at 1 year using the eGFR value closest to 12 months after LUTX and categorized according to KDIGO GFR categories: G1 ≥90, G2 60–89, G3 20–59, G4 15–29, and G5 <15 mL/min/1.73 m^2^ or chronic RRT. Because albuminuria and persistence over ≥3 months were not systematically assessed, this outcome was considered an eGFR-based GFR category at 1 year rather than a definitive CKD diagnosis.

**Sensitivity Analysis**

As a sensitivity analysis, an alternative unsupervised clustering approach (i.e., Hierarchical Cluster Analysis - HCA) was applied to the same four biomarkers used for the latent profile analysis. Biomarker concentrations were log-transformed and standardized before analysis. Hierarchical agglomerative cluster analysis was performed using Ward’s minimum-variance method, with Euclidean distance as the dissimilarity measure. The number of clusters was prespecified at k=3 to permit direct comparison with the three-class LPA solution. Individual cluster assignments obtained by HCA were cross-tabulated against LPA class membership to assess classification agreement. For each method, cluster-specific biomarker centroids were calculated as the mean standardized value of each biomarker within each cluster. Similarity of the biological profiles identified by the two approaches was assessed by Pearson correlation between the corresponding HCA and LPA centroids across the 12 cluster–biomarker combinations.

**Exploratory individual-level renal biomarker phenotype classifier**

To translate the selected latent profile analysis (LPA) solution into an individual-level phenotype assignment procedure, we developed an exploratory classifier based directly on the final fitted Gaussian mixture model, without additional model fitting or incorporation of clinical outcomes. The classifier takes as input the raw 36-hour plasma concentrations of sTNFR1, sTNFR2, midkine, and H-FABP, expressed in the same units used in the study dataset. Each biomarker is natural-log transformed and standardized using the fixed preprocessing parameters applied in the derivation dataset. The resulting four-dimensional standardized biomarker profile is then evaluated against the class-specific distributions estimated by the selected LPA model. Using the fixed class means, covariance structure, and class proportions of the fitted mixture model, the posterior probability of membership in each latent class is calculated for the individual patient. The patient is assigned to the phenotype with the highest posterior probability, while the complete set of posterior probabilities and the corresponding classification uncertainty, defined as 1 minus the maximum posterior probability, are retained. No model parameters are re-estimated when a new patient is classified.

The final three-class LPA solution was implemented in R using the fitted EEI Gaussian mixture model. The numerical mixture components were mapped to the corresponding biomarker-defined phenotypes as follows: class 1, intermediate; class 2, low; and class 3, high. As a technical consistency check, the classifier was applied to the 73 patients included in the original LPA as if they were new observations. Phenotype assignment was concordant with the original LPA classification in all 73 patients. The classifier should therefore be considered a direct implementation of the fitted LPA solution rather than a newly developed AKI prediction model. In particular, the posterior probabilities represent probabilities of membership in the biomarker-defined phenotypes and should not be interpreted as individual probabilities of developing AKI.

For reproducibility, the classifier is provided with the Online Supplement as two files: LUTX_renal_biomarker_phenotype_classifier.R, containing the preprocessing and phenotype-assignment functions, and LUTX_renal_biomarker_phenotype_model.rds, containing the frozen fitted mixture model. Both files should be placed in the same R working directory. After loading the mclust package and sourcing the R script, a new patient can be classified by entering the four raw 36-hour biomarker concentrations into the classify_patient() function. The function returns the posterior probabilities of membership in the low-, intermediate-, and high-biomarker phenotypes, the phenotype corresponding to the highest posterior probability, the maximum posterior probability, and the associated classification uncertainty. The classifier is provided as an exploratory research tool to facilitate reproducibility and future independent validation and is not intended for clinical decision-making.

An example command is shown below:

classify_patient(

stnfr1 = 1.93,

stnfr2 = 0.33,

midkine = 8858.1,

hfabp = 33.97

)

For this illustrative patient, the classifier generates the following output:

Exploratory individual-level phenotype classifier

-------------------------------------------------

Phenotype Posterior_probability

Low	0.1046

Intermediate	0.8954

High	0.0001

Assigned phenotype: Intermediate

Maximum posterior probability: 0.8954

Classification uncertainty: 0.1046

Representative R console output generated by the classifier after entry of the four 36-hour plasma biomarker concentrations. The classifier provides the posterior probabilities of membership in the low-, intermediate-, and high-biomarker phenotypes and assigns the patient to the phenotype with the highest posterior probability. The maximum posterior probability and classification uncertainty, defined as 1 minus the maximum posterior probability, are also reported. Posterior probabilities refer to membership in the biomarker-defined phenotypes and do not represent calibrated individual probabilities of AKI. The example illustrates individual-level implementation of the fitted LPA model; the classifier has not undergone external validation and is intended for research use only.

**Additional results**

**Figure S1. Patients enrollment flowchart**

**Table S1. Clinical Outcomes stratified by Acute Kidney Injury (AKI) stage**

| **Outcome** | **Overall (n=84)** | **AKI=0 (n=54)** | **AKI>0 (n=30)** | **p** | **OR [95% CI] or**  **Effect Size §** |
| --- | --- | --- | --- | --- | --- |
| AKD >0, n | 62 (74%) | 33 (62%) | 29 (97%) | <0.001 | 17.5 [2.21-139.00] |
| CKD >0, n | 77 (92%) | 49 (91%) | 28 (93%) | 0.675 | 1.42 [0.28-10.4] |
| PGD grade 3 at 72 hours, n | 12 (14%) | 2 (3%) | 10 (33%) | <0.001 | 13.0 [2.61-64.60] |
| IMV length, days | 1 [1-4] | 1 [1-2] | 4 [2-19] | <0.001 | 0.159 |
| 28-Days IMV-free days | 27 [23-27] | 27 [26-27] | 23 [0-26] | <0.001 | 0.425 |
| 28-days organ support-free days | 26 [23-27] | 27 [25-27] | 23 [0-26] | <0.001 | 0.416 |
| 28-days ICU-free days | 24 [21-25] | 25 [24-26] | 16 [0-26] | <0.001 | 0.444 |
| 60-days hospital-free days | 32 [9-41] | 35 [26-41] | 8 [0-39] | 0.001 | 0.396 |

AKI, Acute Kidney Injury; AKD, Acute Kidney Disease; PGD, Primary Graft Dysfunction; IMV, Invasive Mechanical Ventilation; ICU, Intensive Care Unit; OR, Odds Ratio; CI, Confidence Interval. §) Vargha and Delaney's A or Cohen's D Effect Size of the AKI>0 vs. AKI=0 cohort.

**Figure S2. Distribution of Acute Kidney Disease (AKD) Stages and Primary Graft Dysfunction (PGD) Grades According to Acute Kidney Injury (AKI) Stage.**

Mosaic plot showing the proportion of patients in each AKD stage (0–3) (left) and in each PGD grade at 72 hours (0–3) (right) within each AKI stage (0–3). The width of each column is proportional to the number of patients in the corresponding AKI stage, and the height of each colored segment represents the percentage of patients classified in each AKD stage and PGD grade.

**Figure S3. Kaplan–Meier Survival Curves According to Acute Kidney Injury (AKI) Status after Lung Transplantation (LUTX).**

Survival probability over time after LUTX is shown for patients without AKI (AKI = 0) and for those who developed AKI (AKI > 0). The x-axis indicates days from transplantation and the y-axis shows cumulative survival.

**Table S2. Performance Metrics** **of u[TIMP-2]*[IGFBP-7] in Predicting Acute Kidney Injury (AKI) >0**.

|  | **u[TIMP-2]*[IGFBP-7]**  **> 0.3 (ng/mL)²/1000** | |
| --- | --- | --- |
|  | **6 hours** | **36 hours** |
| Prevalence | 0.35 [0.28-0.43] | 0.36 [0.29-0.43] |
| Sensitivity | 0.33 [0.22-0.45] | 0.48 [0.35-0.60] |
| Specificity | 0.81 [0.73-0.87] | 0.74 [0.65-0.81] |
| PPV | 0.50 [0.35-0.64] | 0.51 [0.38-0.64] |
| NPV | 0.68 [0.60-0.76] | 0.71 [0.62-0.79] |
| Positive LR | 1.80 [1.05-3.06] | 1.89 [1.23-2.89] |
| Negative LR | 0.81 [0.66-0.99] | 0.69 [0.52-0.91] |

TIMP-2, Urinary Tissue Inhibitor of Metalloproteinases-2; IGFBP-7, Insulin-Like Growth Factor Binding Protein-7; PPV, Positive Predictive Value; NPV, Negative Predictive Value; LR, Likelihood Ratio.

**Figure S4.** **Receiver Operating Characteristic (ROC) Curves of u[TIMP-2]*[IGFBP-7] for Prediction of Acute Kidney Injury (AKI).**

 ROC for u[TIMP-2]*[IGFBP-7] measured 6 hours after reperfusion (left) and 36 hours after reperfusion (right) for AKI (AKI>0). TIMP-2, Urinary Tissue Inhibitor of Metalloproteinases-2; IGFBP-7, Insulin-Like Growth Factor Binding Protein-7.

**Table S3. Clinical Outcomes Stratified by Subclinical Acute Kidney Injury (AKI) strata**

|  | **AKI=0** | **subclinical AKI** | **AKI>0** | **p*** |
| --- | --- | --- | --- | --- |
| *KIDGO criteria* | **no** | | **yes** |  |
| *u[TIMP-2]*[IGFBP-7] > 0.3 (ng/mL)²/1000* | **no** | **yes** |  |  |
| **n** | 27 | 26 | 30 |  |
| AKD >0 | 18 (66%) | 15 (57%) | 29 (97%) | 0.381 |
| CKD >0 | 24 (88%) | 23 (92%) | 28 (93%) | 0.967 |
| PGD grade 3 (72 hrs) | 0 (0%) | 2 (7%) | 10 (33%) | 0.087 |
| IMV, days | 1 [1-2] | 1 [1-4] | 4 [2-19] | 0.099 |
| 28-Days IMV-free days | 27 [26-27] | 27 [24-27] | 23 [0-26] | 0.099 |
| 28-days organ support-free days | 27 [26-27] | 26 [23-27] | 23 [0-26] | **0.042** |
| 28-days ICU-free days | 25 [25-26] | 24 [22-25] | 16 [0-24] | 0.136 |
| 60-days Hospital-free days | 38 [32-42] | 31 [22-40] | 8 [0-39] | 0.149 |

TIMP-2, Urinary Tissue Inhibitor of Metalloproteinases-2; IGFBP-7, Insulin-Like Growth Factor Binding Protein-7; AKI, Acute Kidney Injury; AKD, Acute Kidney Disease; PGD, Primary Graft Dysfunction; IMV, Invasive Mechanical Ventilation; ICU, Intensive Care Unit. *) AKI=0 vs. subclinical AKI.

**Table S4. Biomarkers Concentration Stratified by Timepoint and Acute Kidney Injury (AKI) Strata**

| **Time from reperfusion** | **biomarker** | **AKI=0** | **AKI>0** | **p** |
| --- | --- | --- | --- | --- |
| 6 hours | sTNFR1 (ng/mL) | 1.72 [13125-2055] | 1.99 [1335-2445] | 0.158 |
|  | sTNFR2 (ng/mL) | 0.44 [0.3325-0.59] | 0.44 [0.25-0.785] | 0.261 |
|  | midkine (ng/mL) | 7.39 [5.46-11.67] | 9.73 [6.47-13.14] | 0.129 |
|  | H-FABP (ng/mL) | 35.40 [25.37-56.57] | 42.94 [33.44-67.59] | 0.149 |
| 36 hours | sTNFR1 (ng/mL) | 1.66 [1.37-2.13] | 2.58 [1.88-3.48] | **0.002** |
|  | sTNFR2 (ng/mL) | 0.37 [0.27-0.63] | 0.65 [0.48-0.99] | **0.004** |
|  | midkine (ng/mL) | 11.99 [5.41-19.42] | 12.76 [8.24-30.55] | 0.095 |
|  | H-FABP (ng/mL) | 15.71 [10.78-23.47] | 34.84 [25.51-49.96] | **<0.001** |

Soluble Tumor Necrosis Factor Receptors 1 and 2 (sTNFR1, sTNFR2); Heart-Type Fatty Acid–Binding Protein, H-FABP; AKI, Acute Kidney Injury.

**Figure S5. Receiver Operating Characteristic (ROC) Curve of Soluble Tumor Necrosis Factor Receptors 1 and 2 (sTNFR1, sTNFR2), midkine, and Heart-Type Fatty Acid–Binding Protein (H-FABP)** **for prediction of Acute Kidney Injury (AKI).**

From left to right: ROC for sTNFR1, sTNFR2, midkine, and H-FABP measured 6 hours after reperfusion (upper row) and 36 hours after reperfusion (lower row) for AKI (AKI>0).

**Table S5. Latent Profile Analysis Model Fit at 6 Hours**

| **K** | **logLik** | **AIC** | **BIC** | **entropy** | **min** | **class sizes** |
| --- | --- | --- | --- | --- | --- | --- |
| 4 | -328.130308 | 702.260 | -755.254 | 0.778 | 2 | 41 / 14 / 17 / 2 |
| 2 | -353.715998 | 733.431 | -763.384 | 0.999 | 1 | 73 / 1 |
| 3 | -347.235305 | 730.470 | -771.943 | 0.996 | 1 | 71 / 1 / 2 |
| 1 | -382.035169 | 780.070 | -798.502 |  | 74 | 74 |

Comparison of latent profile analysis (LPA) models with 1 to 4 classes using biomarker measurements obtained 6 hours after reperfusion. For each model, the table reports the number of classes (K), log-likelihood (logLik), Akaike Information Criterion (AIC), Bayesian Information Criterion (BIC), entropy, minimum class size (min), and class sizes. Lower AIC and BIC values indicate better relative model fit, whereas higher entropy indicates greater classification certainty.

**Table S6. Latent Profile Analysis Model Fit at 36 Hours**

| **K** | **logLik** | **AIC** | **BIC** | **entropy** | **min** |
| --- | --- | --- | --- | --- | --- |
| 1 | -424.813 | 865.6265741 | -883.950 |  | 73 |
| 2 | -392.197 | 810.3949938 | -840.170 | 0.835 | 17 |
| **3** | **-375.076** | **786.1530688** | **-827.381** | **0.804** | **14** |
| 4 | -369.320 | 784.6411334 | -837.321 | 0.781 | 13 |

Comparison of latent profile analysis (LPA) models with 1 to 4 classes using biomarker measurements obtained 36 hours after reperfusion. For each model, the table reports the number of classes (K), log-likelihood (logLik), Akaike Information Criterion (AIC), Bayesian Information Criterion (BIC), entropy, minimum class size, and class sizes. Lower AIC and BIC values indicate better relative model fit, whereas higher entropy indicates greater classification certainty.

**Table S7. Patients' Characteristics Stratified by Latent Biological Classes Based on 36-Hour Point-Of-Care Biomarkers**

|  |  | | | | **Low-risk**  **N=22** | | **Intermediate-risk**  **N=37** | | **High-risk**  **N=14** | | **p** | |
| --- | --- | --- | --- | --- | --- | --- | --- | --- | --- | --- | --- | --- |
| Enlistment | Female gender | | | | 10 (45%) | | 16 (43%) | | 5 (35%) | | 0.837 | |
|  | Age at LUTX (years) | | | | 52 [40-60] | | 59 [50-64] | | 53 [38-61] | | 0.114 | |
|  | BMI (kg/m^2^) | | | | 23.4 [17.8-27.5] | | 23.5 [20.2-27.1] | | 23.4 [18.6-25.0] | | 0.627 | |
|  | Diagnosis | Interstitial Lung Disease | | 12 (54%) | | 22 (59%) | | 6 (42%) | | 0.686 | |  |
|  |  | CF/Bronchiectasis | | 6 (27%) | | 9 (24%) | | 3 (21%) | |  |  |  |
|  |  | COPD | | 3 (13%) | | 3 (6%) | | 3 (21%) | |  |  |  |
|  |  | Other | | 1 (5%) | | 3 (8%) | | 1 (7%) | |  |  |  |
|  |  | Vascular | | 0 (0%) | | 0 (0%) | | 1 (7%) | |  |  |  |
|  | Comorbidities | | Hypertension | | 7 (31%) | | 12 (32%) | | 5 (35%) | | 0.968 | |
|  |  |  | Diabetes | | 1 (5%) | | 5 (13%) | | 5 (35%) | | **0.045** | |
|  |  |  | CKD* | | 1 (5%) | | 10 (27%) | | 5 (35%) | | **0.027** | |
|  |  |  | Hepatopathy | | 2 (9%) | | 1 (3%) | | 0 (0%) | | 0.297 | |
|  | Lung Allocation Score | | | | 41.5 [36.8-46.0] | | 41.5 [35.9-53.3] | | 43.4 [37.8-47.4] | | 0.729 | |
| Donor | Age (years) | | | | 55 [35-58] | | 52 [32-58] | | 56 [36-6] | | 0.751 | |
|  | OTO Score** | | | | 4 [2-5] | | 3 [1-4] | | 3 [1-5] | | 0.287 | |
|  | Cold Ischemia Time (min) | | | | 415 [383-527] | | 467 [362-692] | | 603 [429-755] | | 0.185 | |
|  | Warm Ischemia Time (min) | | | | 70.2 [66.4-81.7] | | 75.0 [64.5-88.7] | | 77.0 [60.6-86.4] | | 0.834 | |
|  | DCD (y/n) | | | | 3 (21%) | | 7 (50%) | | 4 (28%) | | 0.587 | |
|  | Induction Therapy | | | | 19 (86%) | | 33 (89%) | | 11 (78%) | | 0.616 | |
| Surgical | EVLP (y/n) | | | | 5 (23%) | | 8 (22%) | | 4 (28%) | | 0.873 | |
|  | RBC (units) | | | | 3 [2-6] | | 4 [2-6] | | 8 [3-11] | | 0.187 | |
|  | FFP (units) | | | | 2 [0-5] | | 4 [2-6] | | 4 [2-8] | | 0.227 | |
|  | Blood Components (L) | | | | 2.15 [0.61-3.86] | | 2.60 [1.45-3.75] | | 4.05 [2.15-7.25] | | 0.145 | |
|  | Intraoperative ECMO (y/n) | | | | 11 (50%) | | 19 (51%) | | 9 (64%) | | 0.656 | |

Categorical variables are presented as counts and percentages (n, %), while continuous variables are expressed as medians with interquartile ranges.

Abbreviations. BMI: body mass index; CF: Cystic Fibrosis; COPD: Chronic Obstructive Pulmonary Disease; DCD, donor after cardiac death; EVLP, Ex-Vivo Lung Perfusion; RBC, red blood cell; FFP, fresh frozen plasma; ECMO, extracorporeal membrane oxygenation; NA, not applicable.

Notes. ** Not calculated for graft undergone EVLP.

**Figure S6 Six-month clinical status stratified by phenotype**Mosaic plot showing the distribution of 6-month clinical status across the three kidney-injury risk classes identified by latent profile analysis (low-risk, n=22; intermediate-risk, n=37; high-risk, n=14). The width of each column is proportional to class size, and the height of each segment reflects the proportion of patients within each class who were rejection-free alive, experienced rejection, or died by 6 months.

**Figure S7. Hierarchical Cluster Analysis of plasma biomarker profiles at 36 hours after graft reperfusion.**

Heatmap and dendrogram showing hierarchical agglomerative clustering of patients with complete biomarker data (n=73), based on log-transformed and standardized plasma concentrations of sTNFR1, sTNFR2, midkine, and H-FABP measured 36 hours after graft reperfusion. Clustering was performed using Ward’s minimum-variance method with Euclidean distance. Each row represents an individual patient and each column a biomarker; colors indicate relative biomarker concentrations on the standardized scale (blue, lower; red, higher). Soluble Tumor Necrosis Factor Receptors 1 and 2, STNFR1 and STNFR2; Heart-Type Fatty Acid–Binding Protein, H-FABP.

**Table S8. Biomarker Concentrations Stratified by Hierarchical Cluster Analysis Phenotypes at 36 Hours After Reperfusion**

|  | **Low-risk**  **N=14** | **Intermediate-risk**  **N=43** | **High-risk**  **N=16** | **p** |
| --- | --- | --- | --- | --- |
| **sTNFR1 (ng/mL)** | 1.38 [1.11-1.69] | 1.81 [1.45-2.17]^1^ | 3.49 [3.27-4.27]^1,2^ | <0.001 |
| **sTNFR2 (ng/mL)** | 0.23 [0.17-0.35] | 0.47 [0.35-0.64]^1^ | 1.14 [0.95-1.66]^1,2^ | <0.001 |
| **midkine (ng/mL)** | 4.51 [3.42-5.47] | 14.39 [9.41-30.63]^1^ | 14.17 [9.87-22.91]^1^ | <0.001 |
| **H-FABP (ng/mL)** | 13.08 [9.94-18.09] | 19.13 [11.46-29.09]^1^ | 50.36 [31.06-67.59]^1,2^ | <0.001 |

Plasma biomarker concentrations measured 36 hours after reperfusion according to the three phenotypes identified by Hierarchical Cluster Analysis (low-, intermediate-, and high-risk). Data are reported as median [interquartile range]. P values refer to the overall comparison across classes. Superscript numbers indicate significant pairwise differences: 1 vs low class; 2 vs intermediate class. sTNFR1, Soluble Tumor Necrosis Factor Receptor 1; sTNFR2, Soluble Tumor Necrosis Factor Receptor 2; H-FABP, Heart-Type Fatty Acid-Binding Protein.

**Figure S8. Distribution of Acute Kidney Injury (AKI) stages across phenotypes at 36 hours after reperfusion.**

Mosaic plot showing the distribution of Acute Kidney Injury (AKI) stages (0–3) across the three Hierarchical Cluster Analysis phenotypes identified from plasma biomarker measurements obtained 36 hours after reperfusion (low, intermediate, and high). The width of each column is proportional to the number of patients in the corresponding phenotype, and the height of each colored segment represents the proportion of patients within each AKI stage. Percentages are reported within each class.
